# On the Treatment of Sprains of the Ankle

**Published:** 1851-01

**Authors:** 


*On the Treatment of Sprains of the Ankle.* By M. Baudens.—M. Baudens observes, that judging by the frequency of the occurrence of this accident, its treatment ought to be well understood and successfully practised; but that this is in fact far from being the case, and he is therefore desirous of making his own plan of treating it, by the cold-bath and gum bandage, more extensively known.

The indications are, first, to prevent or remove inflammation, and then to secure immovability to the distended or lacerated parts, until they have recovered their power, the patient being at the same time allowed the use of the limb. For the purpose of subduing inflammation, numbers of leeches are usually applied, and then an emollient cataplasm; and M. Baudens feels convinced that it is in consequence of such treatment that degenerated sprains so often augment the number of amputations in hospitals. By free leeching of a joint, the seat of sprain, two mischievous effects are produced. In the first place, the pain, which is the first of the series of inflammation after sprain, is increased by the leech-bites, in place of being mitigated; and, in the next, the increased afflux of blood towards the part is encouraged instead of being repelled. M. Baudens, on these grounds, strictly forbids the application of leeches in all surgical maladies attended with acute inflammation, while he often derives most excellent aid from their employment in chronic inflammations; thus, by the induction of a temporary congestion, giving a fillip to the too languid action of the part. When blood need be taken in sprain, he abstracts it by venesection, although probably both the profession and the public, from the force of habit, would tax with ignorance any one who neglected the use of leeches. As to emollient cataplasms, they favor in place of opposing the afflux of fluids to the parts, while the long maceration the joint has been thus submitted to, deprives it of its elasticity, gives rise to a pasty *engorgement*, and predisposes to the formation of white swelling.

M. Baudens has pursued his own plan of treatment now for twenty years, and under it his patients have been enabled to resume their trying military duties in a very short time. He is not the first who has employed cold water in the treatment of sprain; but his originality consists in trusting to it alone, and continuing its application for so long a period. His plan of employing it, contrasted with that of his predecessors, may be thus summed up:—1. *Period of the Application.* Cold has usually been thought desirable only when it could be resorted to very shortly after the accident; but he applies it not only immediately, but also several hours or days after the occurrence, or even in chronic sprain—whenever, in fact, there is a *morbid degree of heat to abstract.* 2. The local bath has never been ordered by others for longer than five or six hours, although some practitioners, since his first publication on the subject, have ventured to extend it to twenty-four. In certain of his cases, however, immersion has been continued for eight or ten days, and, in one example, for fourteen days; while in no case has it been less than for two. 3. *Mode of Application.*—The vessel containing the water is brought to the bedside of the patient, so that he can conveniently place his leg in it, having the heel resting on a sponge at the bottom, the leg and thigh being supported by cushions, so that the position may be maintained as many days as required. In the vessels used at the Val-de-Grace the water reaches as high as the middle of the leg, and is changed about every three hours in order to keep it sufficiently cool. Spring water is usually employed, and if the inflammation is intense, ice is added. A purgative is given, and, if indicated, one or two bleedings are resorted to. 4. *Effects.*—One of the first of these is the cessation of pain, which sometimes occurs at once, and at others in an hour or two. From the moment the foot is placed in the bath, the swelling becomes stationary, and soon after, with the heat and redness, decreases. About the fourth or fifth day the part becomes wrinkled like the hands of a washerwoman; and usually about the third or fourth day, the patient finds the water too cold, and then the limb is removed from it—the period for doing this being regulated by the patient, he being told to keep it in only as long as he derives comfort from so doing. Few of the patients suffer from any general reaction. *Gangrene* has been said to have resulted from this application, but the author has never met with such a case. The patient sometimes persists in keeping the limb in water after the dispersion of the heat and pain, and the consequence is the production of engorgement of the joint, a tense state and dark color of the skin, together sometimes with darkish lines—precursory signs of congelation in fact—on seeing which the joint should be enveloped in a fomentation of elder-flowers and poppy-heads at the temperature of the atmosphere. The objections which have been urged from the fear of producing *repercussion*, are quite theoretical and unfounded. It is in fact only the *excess* of *morbid caloric* that is abstracted.

*Gum-bandage.*—When the inflammation has been subdued, all the depressions in the vicinity of the joint are filled with wadding, and a bandage carefully and equably applied. This is well moistened, by means of a brush with very thick gum, which in a short time imparts to it almost the hardness of wood. After this has been worn for twenty-five or thirty days, it is removed, and the joint slowly and gradually exercised; for want of which precaution many patients (especially those treated by leeches and poultices) suffer all the symptoms of a sub-inflammation of the white tissues of the joints, even for years.—*Gaz. des. Hop.*, 1850, Nos. 5 and 6.

